# Glioblastoma treatment patterns and outcomes over 18 years in an Irish cancer centre

**DOI:** 10.1007/s11845-026-04449-1

**Published:** 2026-06-05

**Authors:** Yazan H. Mustafa, Rachel J. Keogh, Simon L. Barry, Bryan Dillon, Wail Mohammed, Chris Lim, Michael Jansen, Niamh Bermingham, Martin Higgins, Seamus O’Reilly, Jack P. Gleeson, Richard M. Bambury

**Affiliations:** 1 School of Medicine, University College Cork, Cork, Ireland; 2 Department of Medical Oncology, CUH/UCC Cancer Centre, Cork University Hospital, Cork, Ireland; 3 Cancer Research @UCC, College of Medicine and Health, University College Cork, Cork, Ireland; 4 Health Service Executive, Dublin, D08 W2A8 Ireland; 5 Department of Neurosurgery, CUH/UCC Cancer Centre, Cork University Hospital, Cork, Ireland; 6 Department of Neuropathology, CUH/UCC Cancer Centre, Cork University Hospital, Cork, Ireland; 7 Department of Radiation Oncology, CUH/UCC Cancer Centre, Cork University Hospital, Cork, Ireland; 8 Cancer Trials Ireland, Dublin, Ireland

**Keywords:** Chemotherapy, Glioblastoma, Overall Survival, Radiation, Stupp Protocol

## Abstract

**Background:**

Glioblastoma is the most common malignant brain tumour in adults and carries a poor prognosis despite advances in molecular characterisation and therapy. The 2021 World Health Organisation classification redefined glioblastoma as an IDH-wildtype diffuse astrocytic tumour with aggressive histological or molecular features. Standard of care therapy has changed little over the last two decades, and outcomes remain suboptimal, with considerable heterogeneity in survival influenced by clinical, molecular, and treatment-related factors.

**Aims:**

This study evaluates the clinical and molecular characteristics, treatment strategies, and survival outcomes for glioblastoma patients at Cork University Hospital from 2006–2023 to assess changes in management and outcomes.

**Methods:**

494 patients were diagnosed with glioblastoma between 1st of January 2006 and 31st of December 2023. IDH mutant cases were excluded. Data was extracted from medical records, with survival analyses using the Kaplan–Meier method and COX proportional hazards model.

**Results:**

The median age was 63 years (range: 17–86). Increased availability of molecular markers was observed between the time periods. Median overall survival remained stable over time (2006–2010: 9.9 months; 2011–2014: 11.9 months; 2015–2019: 10.9 months; 2020–2023: 11.1 months), with no statistical significance between periods. On multivariate analysis, key prognostic factors included age at diagnosis, ECOG performance status, glucocorticoid use at baseline, MGMT methylation status, and completion of the adjuvant chemo-radiation.

**Conclusions:**

Despite advances in our understanding of the pathogenesis of glioblastoma, the mOS median overall survival in our real-world patient cohort did not improve over time. The findings emphasise the need for ongoing research efforts to improve outcomes in this lethal disease.

## Background

Glioblastoma is the most common malignant brain tumour in adults and constitutes 51% of all malignant brain tumours and 14% of all brain tumours [1]. Despite advances in understanding the molecular pathogenesis and biology of this tumour [2], the prognosis for patients diagnosed with glioblastoma remains poor with a reported median overall survival (mOS) of 16—22 months for clinical trial participants [3–5] and 6—13 months in real-world populations [6–12]. The global cancer burden of this, and other brain tumours has increased by over 100% in the past 30 years and is projected to increase further between now and 2050 [13, 14].

The most widely used system for the diagnosis and nomenclature of glioma is the World Health Organisation (WHO) classification system. This was updated in 2021 to exclude isocitrate dehydrogenase (IDH) mutated tumours from the glioblastoma category due to their comparatively favourable prognosis, as well as rename the nomenclature of this tumour from glioblastoma multiforme to glioblastoma [15]. According to the 2021 WHO Classification of Tumours of the Central Nervous System, a diagnosis of glioblastoma requires the presence of an IDH-wildtype diffuse astrocytic tumour that either exhibits histopathological features of necrosis and/or microvascular proliferation, or harbours molecular alterations such as TERT promoter mutation, EGFR gene amplification, or combined whole chromosome 7 gain and chromosome 10 loss [15]. These features distinguish glioblastoma from lower-grade IDH-mutant astrocytoma and underpin its aggressive behaviour. However, as previous trials and data were predominantly based on histological diagnosis rather than molecular features, translating from previous trials to current treatment options for patients is not without its controversies.

The prognosis of patients is highly variable, with established prognostic factors including age, Eastern Cooperative Oncology Group (ECOG) performance status, extent of surgical resection, neutrophil–lymphocyte ratio [16] and the need for glucocorticoid use at baseline [17]. Additionally, Hegi et al. demonstrated that O6-methylguanine-DNA-methyltransferase (MGMT) promoter methylation is associated with a 40% relative improvement in mOS for glioblastoma patients treated with Temozolomide [18].

The current standard of care management strategy for glioblastoma has remained largely unchanged for nearly twenty years, following the landmark clinical trial by Stupp et al. [19]. This involves maximal safe surgical resection followed by six weeks of localised radiotherapy with concomitant Temozolomide chemotherapy followed by adjuvant Temozolomide [19–22]. Following progression after first-line therapy, second and third-line treatments are less standardised and options include systemic therapy with Bevacizumab and Lomustine and re-irradiation [23, 24].

Tumour treating fields (TTF) have been incorporated into clinical practice for both newly diagnosed and recurrent glioblastoma cases in some institutions [25, 26] and gained Food and Drug Administration (FDA) approval in 2015 [5]. A meta-analysis of comparative TTF studies indicates that adding tumour treating fields to the first line standard of care Stupp protocol may increase mOS for patients with newly diagnosed glioblastoma by 5 months [5]. Nonetheless, debate persists regarding the optimal application of TTF and its practicality and wider global adaption has been limited [25].

Despite this first-line treatment strategy, disease progression is inevitable in most cases and frequently manifesting an aggressive course with poor prognosis [8, 9, 11, 12, 23, 27–29].

The primary aim of this study was to present the clinical and molecular attributes, therapeutic interventions, and survival outcomes among patients diagnosed with glioblastoma at our institution over an 18-year period from 2006 to 2023, with the goal of guiding further treatment planning and service delivery, as well as identifying areas for potential improvement to inform service development. The secondary objective was to compare the baseline characteristics, therapeutic interventions, and survival outcomes among patients diagnosed with glioblastoma across four diagnostic periods:2006–2010, 2011–2014, 2015–2019 and 2020–2023.

## Methods

Cork University Hospital is one of two National Cancer Control Programme designated cancer centres providing neurosurgery care in the Republic of Ireland. Since 2004, it has been recognised as a European Society of Medical Oncology designated centre of Integrated Oncology and Palliative Care. In this study, a prospectively maintained database from the Department of Neuropathology at Cork University Hospital was used to identify all patients diagnosed with glioblastoma between 1st of January 2006 and 31st of December 2023.

This study focused exclusively on patients diagnosed with glioblastoma who were subsequently referred for assessment to the medical oncology department at Cork University Hospital. Patients with IDH mutant glioblastoma were excluded from the analysis based on the recently updated WHO classification [15]. Our standard approach for eligible patients who are suitable for systemic therapy includes adjuvant concurrent chemoradiotherapy, followed by consolidation Temozolomide chemotherapy as per the Stupp protocol [19].

Electronic medical records of all patients were reviewed to collect data on patient demographics, tumour characteristics, and treatment-related variables. Extracted information included sex, date of birth, age at diagnosis, date of diagnosis, ECOG performance status, history of seizures, baseline glucocorticoid use (at the time of the first medical oncology consultation), IDH mutation status, MGMT methylation status, extent of surgical resection (biopsy only, partial resection, or gross total resection), receipt of radiation therapy (defined as initiation of radiation, regardless of completion), completion of 6 weeks of adjuvant chemoradiotherapy (Stupp I protocol), completion of 6 cycles of adjuvant chemotherapy (Stupp II protocol), receipt of re-resection at disease progression, receipt of second-line chemotherapy, and overall survival.

Extent of surgical resection was categorised as follows: gross total resection was defined as complete removal of all enhancing tumour on postoperative magnetic resonance imaging (MRI) performed within 72 hours of the primary maximal surgical debulking, as reviewed by the multidisciplinary tumour board; subtotal resection was defined as any maximal safe resection that did not achieve gross total resection; and biopsy was defined as any limited sampling of the lesion performed solely for diagnostic purposes.

The study population was stratified into four time periods according to year of diagnosis: January 1st 2006 to December 31st 2010, January 1st 2011 to December 31st 2014, January 1st 2015 to December 31st 2019, and January 1st 2020 to December 31st 2023.

Baseline patient characteristics of each group were compared across the four cohorts using the Chi-Square test. Overall survival was defined as the interval from histologic diagnosis to death. Censored patients were those who were still alive or lost to follow-up at the time of analysis. A data cut-off date of 12 May 2025 was applied; patients who died on or after 13 May 2025 were recorded as alive at the study end. For patients who were lost to follow-up, details were cross-referenced using the national www.rip.ie website which is used for death notifications in Ireland. If no recorded death was found, the date of the last clinic appointment was used as the censoring date. Uncensored patients were those who had died.

Overall survival was estimated using the Kaplan–Meier method, with comparisons between groups assessed using the log-rank test. The COX proportional hazards model was applied to evaluate the impact of various variables on overall survival in both univariate and multivariate analyses. Data were analysed using IBM statistical package for the social sciences statistics, version 29.0.2.0. Statistical significance was defined as a two-tailed p-value of < 0.05. No adjustment for multiple comparisons was performed. For this analysis, we assumed independence of observations, normal distribution of dependent variables, and homogeneity of variance. Cases with missing data were excluded from the respective analyses.

## Results

A total of 494 patients with glioblastoma were referred to the medical oncology department with 110 cases identified between 2006 and 2010, 92 cases between 2011 and 2014, 121 cases between 2015 and 2019, and 171 cases between 2020 and 2023. The median age at diagnosis for the entire cohort was 63 years (range: 17–86) with a predominantly male population (64%). All patients were assessed for eligibility for adjuvant therapy by the medical oncology department, with suitable candidates commencing adjuvant radiotherapy and Temozolomide as per the Stupp protocol [20].

It is important to note that glucocorticoid use at baseline was unknown for 27 patients, and one patient received a radiological diagnosis of glioblastoma without undergoing surgical intervention. These patients were excluded from the analysis in their respective domains.

Baseline patient characteristics across the two time periods are summarised in Table 1. For all cohorts, there was a male predominance.

**Table 1:** Baseline patient characteristics

	***2006–2010***	***2011–2014***	***2015–2019***	***2020–2023***	
**Age at Diagnosis**					
Median (Range)	61 (17–81)	64 (21–80)	62 (21–81)	65 (20–86)	
**Age at Diagnosis**					*p* = 0.101
> 65 Years Old (%)	41 (37)	40 (43)	48 (40)	87 (51)	
≤ 65 Years Old (%)	69 (63)	52 (57)	73 (60)	84 (49)	
**Sex**					*p* = 0.086
Male (%)	77 (70)	59 (64)	84 (69)	98 (57)	
Female (%)	33 (30)	33 (36)	37 (31)	73 (43)	
**IDH Mutation Status**					
Negative (%)	2 (2)	82 (89)	121 (100)	171 (100)	
Unknown (%)	108 (98)	10 (11)	0 (0)	0 (0)	
**MGMT Status**					*p* < 0.001
Confirmed Methylation (%)	0 (0)	3 (3)	48 (40)	74 (43)	
Not Methylated (%)	1 (1)	5 (5)	65 (54)	96 (56)	
Unknown (%)	109 (99)	84 (91)	8 (6)	1 (1)	
**ECOG at Presentation**					*p* = 0.551
0–1 (%)	83 (75)	68 (74)	79 (65)	121 (71)	
2 (%)	23 (21)	20 (22)	38 (31)	41 (24)	
3–4 (%)	4 (4)	4 (4)	4 (3)	9 (5)	
**Seizure at Presentation**					*p* = 0.263
Yes (%)	14 (13)	14 (15)	26 (21)	34 (20)	
No (%)	96 (87)	78 (85)	95 (79)	137 (80)	
**Glucocorticoid at Baseline**					*p* = 0.283
Yes (%)	56 (53)	36 (43)	65 (57)	89 (55)	
No (%)	50 (47)	47 (57)	50 (43)	74 (45)	
**Extent of Surgical Resection**					*p* < 0.001
Biopsy Only (%)	37 (34)	31 (34)	41 (34)	44 (26)	
Partial Resection (%)	69 (63)	58 (63)	57 (47)	107 (62)	
Gross Total Resection (%)	3 (3)	3 (3)	23 (19)	20 (12)	
**Receipt of Radiation**					*p* = 0.015
Yes (%)	105 (95)	81 (88)	99 (82)	146 (85)	
No (%)	5 (5)	11 (12)	22 (27)	25 (15)	
**Stupp I Complete**					*p* = 0.029
Yes (%)	88 (80)	73 (79)	81 (67)	116 (68)	
No (%)	22 (20)	19 (21)	40 (33)	55 (32)	
**Stupp II Complete**					*p* = 0.747
Yes (%)	26 (24)	22 (24)	35 (29)	47 (27)	
No (%)	84 (76)	70 (76)	86 (71)	124 (73)	
**Receipt of Re-Resection**					*p* = 0.443
Yes (%)	16 (15)	8 (9)	16 (13)	27 (16)	
No (%)	94 (85)	84 (91)	105 (87)	144 (84)	
**Receipt of Second-Line Systemic Therapy**					*p* = 0.016
Yes (%)	19 (17)	29 (32)	42 (35)	55 (32)	
No (%)	91 (83)	63 (68)	79 (65)	116 (68)	
**Second-Line Systemic Therapy**					*p* < 0.001
Bevacizumab (%)	11 (58)	29 (100)	41 (98)	54 (98)	
Procarbazine + Lomustine + Vincristine (%)	8 (42)	0 (0)	1 (2)	0 (0)	
Bevacizumab + Lomustine (%)	0 (0)	0 (0)	0 (0)	1 (2)	

In the earliest cohort (2006–2010), 98% of patients had unknown IDH mutation status, as testing was not routinely performed during this period. From 2011 onward, IDH testing became standard, with 89% of patients tested between 2011–2014 and universal testing was achieved from 2015 onward.

The details of surgical and adjuvant therapies across the four diagnostic periods are summarised in Table 1. Surgical management evolved over time (*p* < 0.001): biopsy-only procedures remained relatively stable (34% in 2006–2010 vs 26% in 2020–2023), whereas the proportion of gross total resections increased from 3 to 12%.

The proportion of patients receiving radiotherapy declined modestly across the cohorts (95% in 2006–2010 vs 85% in 2020–2023; *p* = 0.015).

Overall survival including univariate and multivariate analysis is presented in Table 2. The mOS for the entire cohort was 11.0 months. When stratified by diagnostic period, the mOS was 9.9 months for patients diagnosed in 2006–2010, 11.9 months for 2011–2014, 10.9 months for 2015–2019, and 11.1 months for 2020–2023. There was no statistically significant difference in mOS across the four time periods in univariate analysis.

**Table 2:** Prognostic impact of variables on overall survival

**Variables**	*N*	mOS	Univariate			Multivariate		
			**HR**	**95% CI**	***p***	**HR**	**95% CI**	***p***
**Patient Characteristics**								
**Time Period of Diagnosis**								
2006–2010	110	9.9	1.04	0.81–1.34	0.769			
2011–2014	92	11.9	0.97	0.75–1.25	0.802			
2015–2019	121	10.9	0.96	0.76–1.23	0.757			
2020–2023	171	11.1						
**Sex**								
Male	318	10.6	0.91	0.75–1.10	0.316			
Female	176	11.9						
**Age at Diagnosis**								
> 65	216	7.3	1.69	1.40–2.03	< 0.001	1.31	1.07–1.62	0.010
≤ 65	278	13.4						
**ECOG at Presentation**								
0–1	351	13.6						
2	122	4.5	2.58	2.01–3.19	< 0.001	1.48	1.16–1.89	0.002
3–4	21	2.5	10.01	6.18–16.25	< 0.001	3.15	1.70–5.84	< 0.001
**Seizure at Presentation**								
Yes	88	13.3	0.84	0.66–1.07	0.160			
No	406	10.6						
**Glucocorticoid at Baseline**								
Yes	246	9.9	1.25	1.03–1.51	0.021	1.49	1.21–1.83	< 0.001
No	221	13.3						
**Tumour Characteristics**								
**IDH Status**								
Wild Type	376	11.6	1.09	0.88–1.35	0.436			
Not Specified	118	9.9						
**MGMT Status**								
Methylated	125	13.4						
Not Methylated	167	10.4	1.85	1.44–2.37	< 0.001	1.91	1.47–2.50	< 0.001
Not Specified	202	10.4	1.50	1.18–1.91	< 0.001	2.22	1.28–3.86	0.005
**Treatment**								
**Surgery**								
Biopsy	153	5.8	2.79	1.98–3.93	< 0.001	1.30	0.88–1.92	0.185
Partial Resection	291	12.4	1.55	1.12–2.14	0.008	1.07	0.75–1.52	0.712
Gross Total Resection	49	16.7						
**Radiation**								
Received	431	12.4	0.10	0.07–0.13	< 0.001	0.25	0.17–0.37	< 0.001
Not Received	63	1.5						
**Stupp I**								
Complete	358	13.9	0.21	0.16–0.26	< 0.001	0.56	0.41–0.77	< 0.001
In-Complete	136	3.0						
**Stupp II**								
Complete	130	20.1	0.31	0.25–0.39	< 0.001	0.43	0.34–0.55	< 0.001
In-Complete	364	7.5						
**Re-Resection**								
Performed	67	17.3	0.55	0.42–0.72	< 0.001	0.78	0.57–1.05	0.102
Not Performed	427	9.4						
**Second-Line Chemotherapy**								
Received	145	16.0	0.53	0.44–0.65	< 0.001	0.80	0.63–1.01	0.061
Not Received	349	7.4						

Univariate analysis revealed notable improvements in mOS among specific patient subgroups: patients 65 years and younger (mOS 13.4 months vs. 7.3 months in older patients), patients undergoing gross total resection (mOS 16.7 months vs. 12.4 months for partial resection and 5.8 months for biopsy only), those receiving radiotherapy (mOS 12.4 months vs. 1.5 months without radiotherapy), and patients completing consolidation Temozolomide Stupp I (mOS 13.9 months vs. 3.0 months for incomplete treatment).

**Fig. 1:**
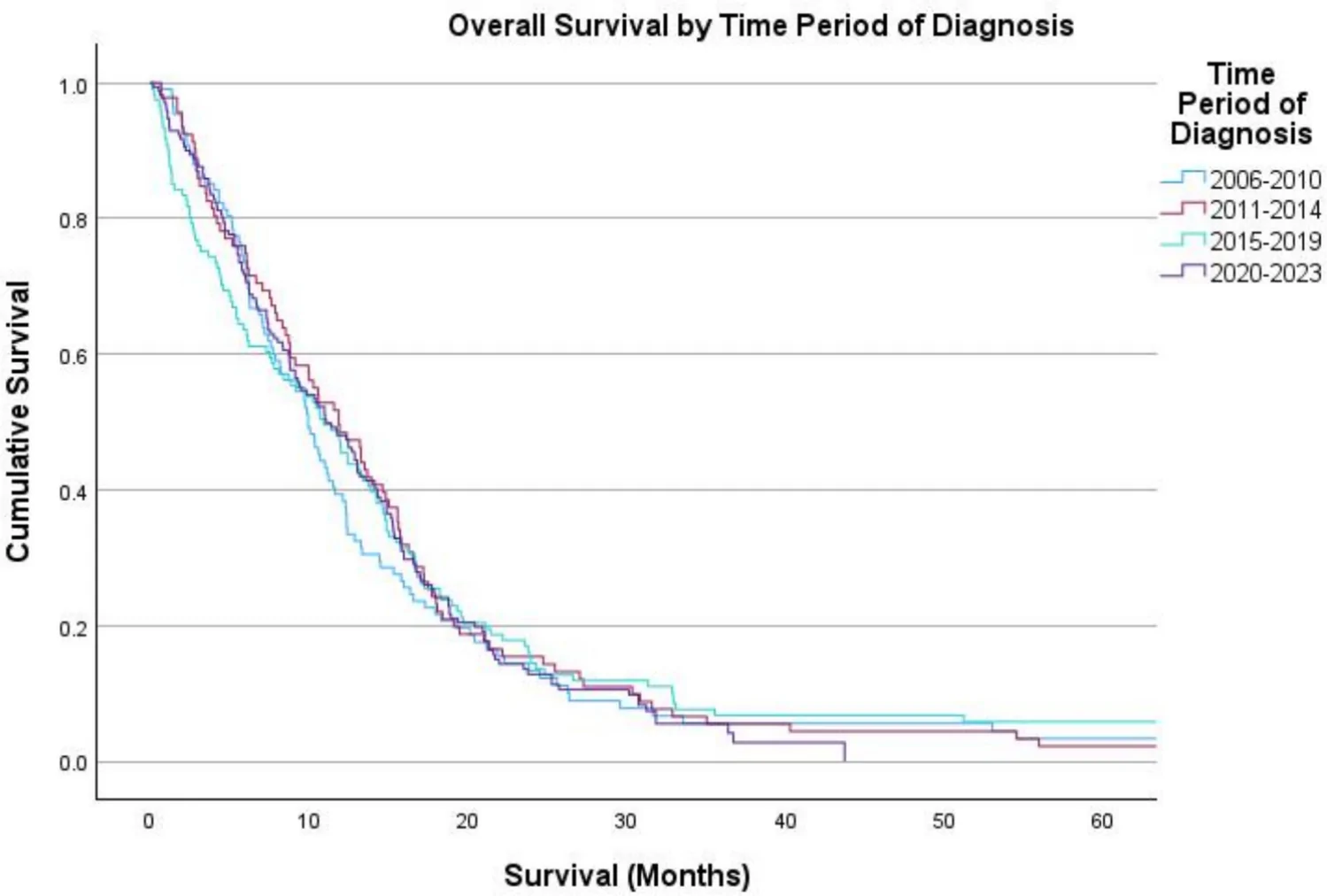
Kaplan–Meier Curve based on time period of diagnosis

**Fig. 2:**
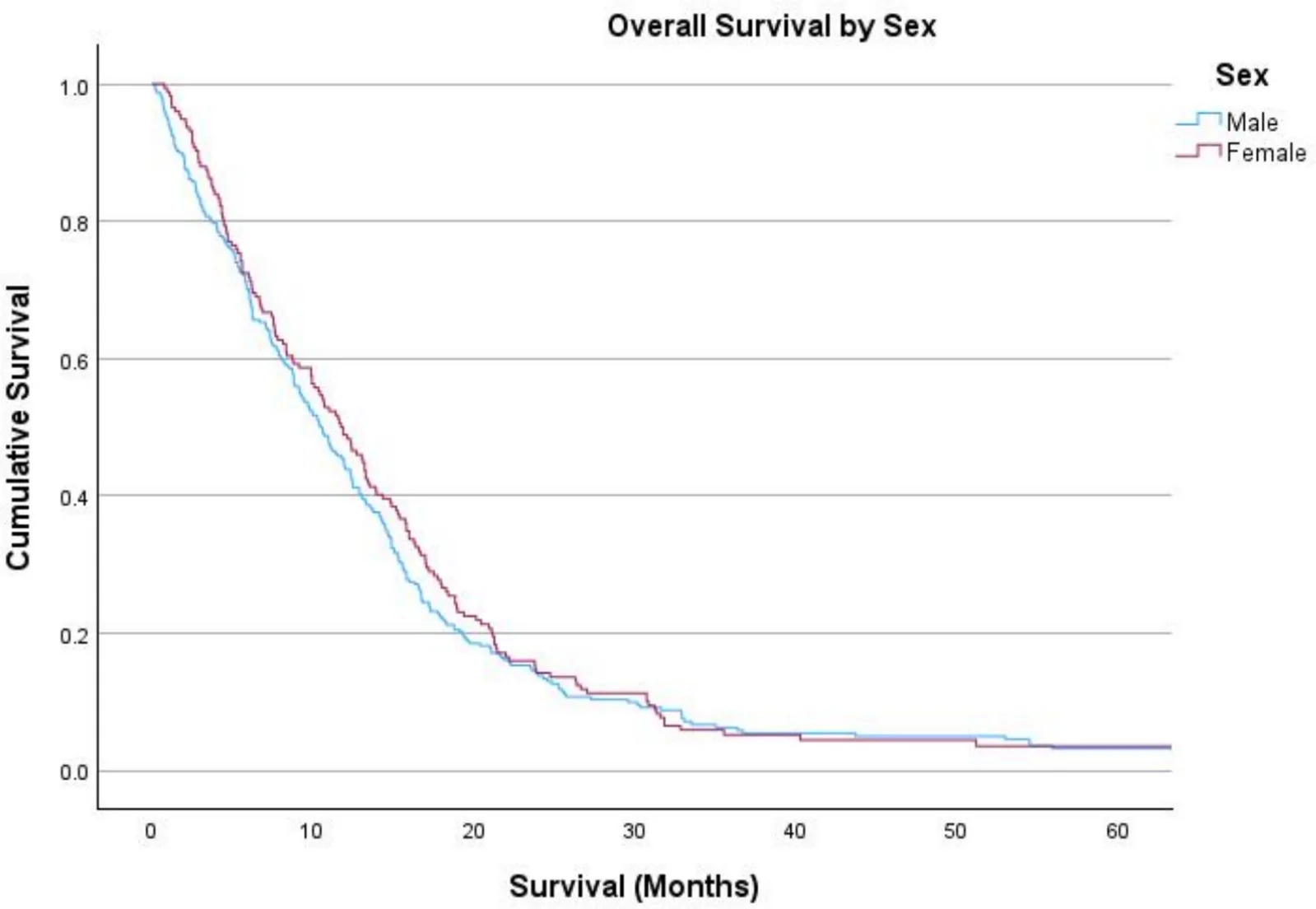
Kaplan–Meier Curve based on sex

**Fig. 3:**
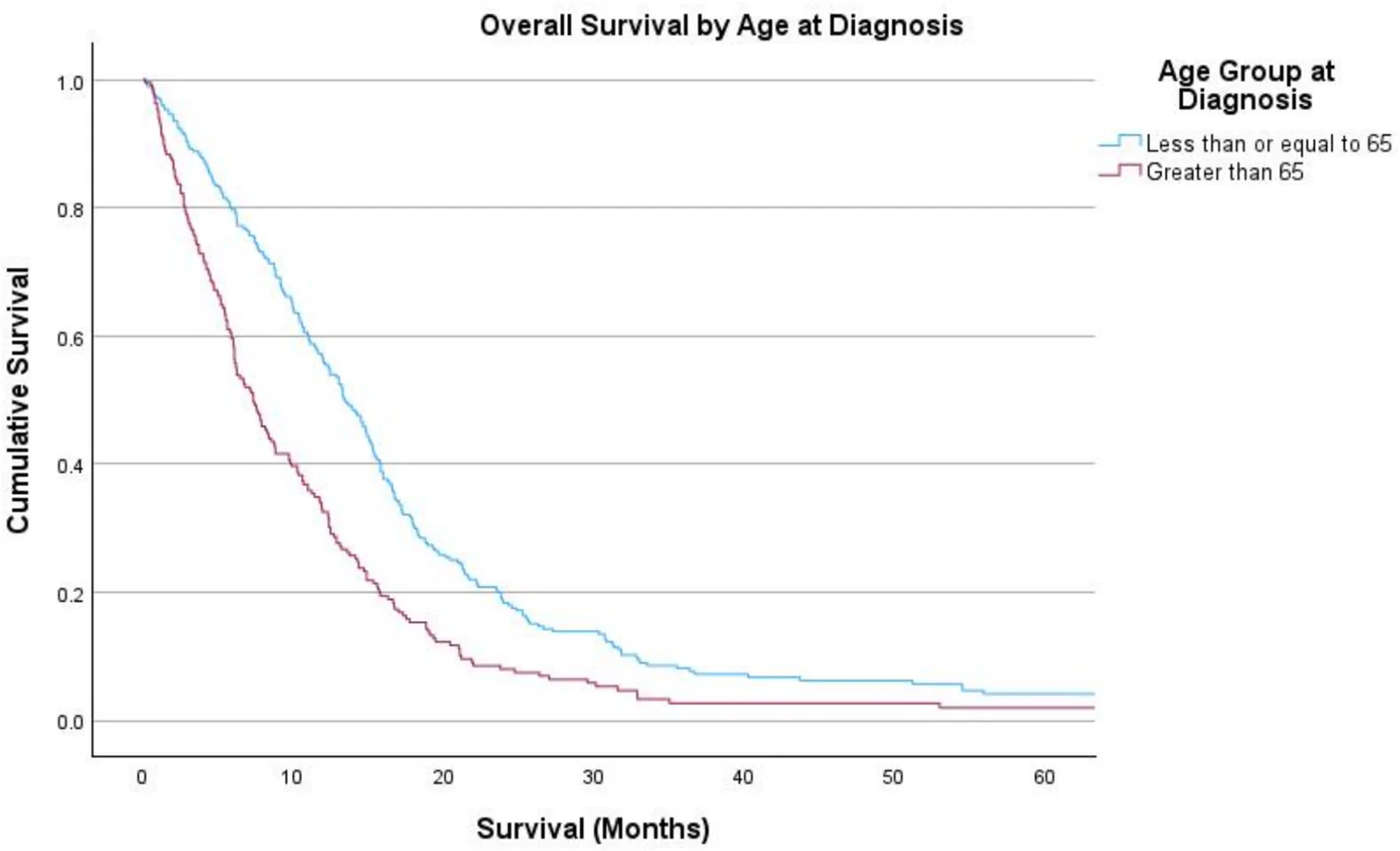
Kaplan–Meier Curve based on age at diagnosis

**Fig. 4:**
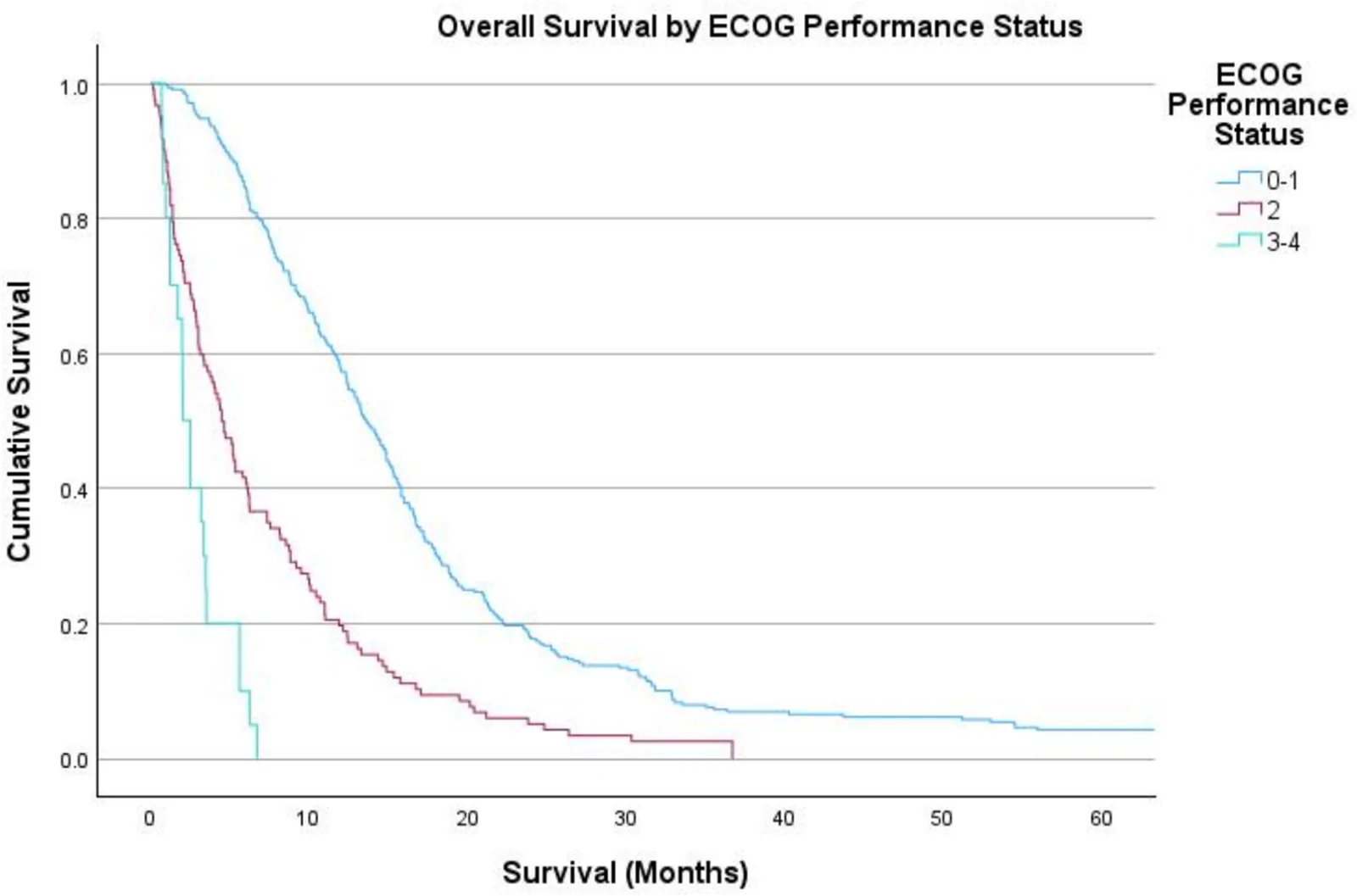
Kaplan–Meier Curve based on ECOG performance status

**Fig. 5:**
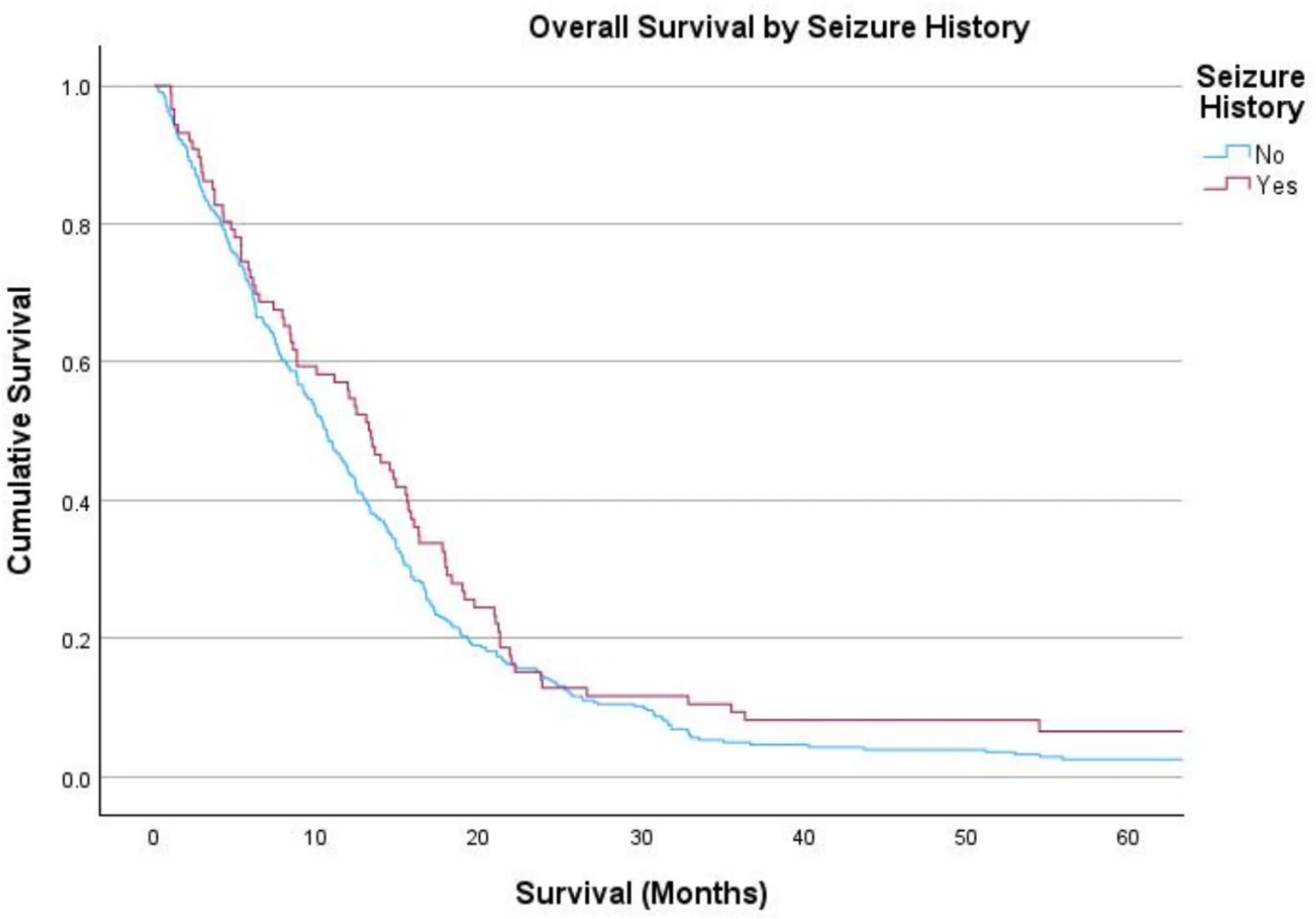
Kaplan–Meier Curve based on seizure history

**Fig. 6:**
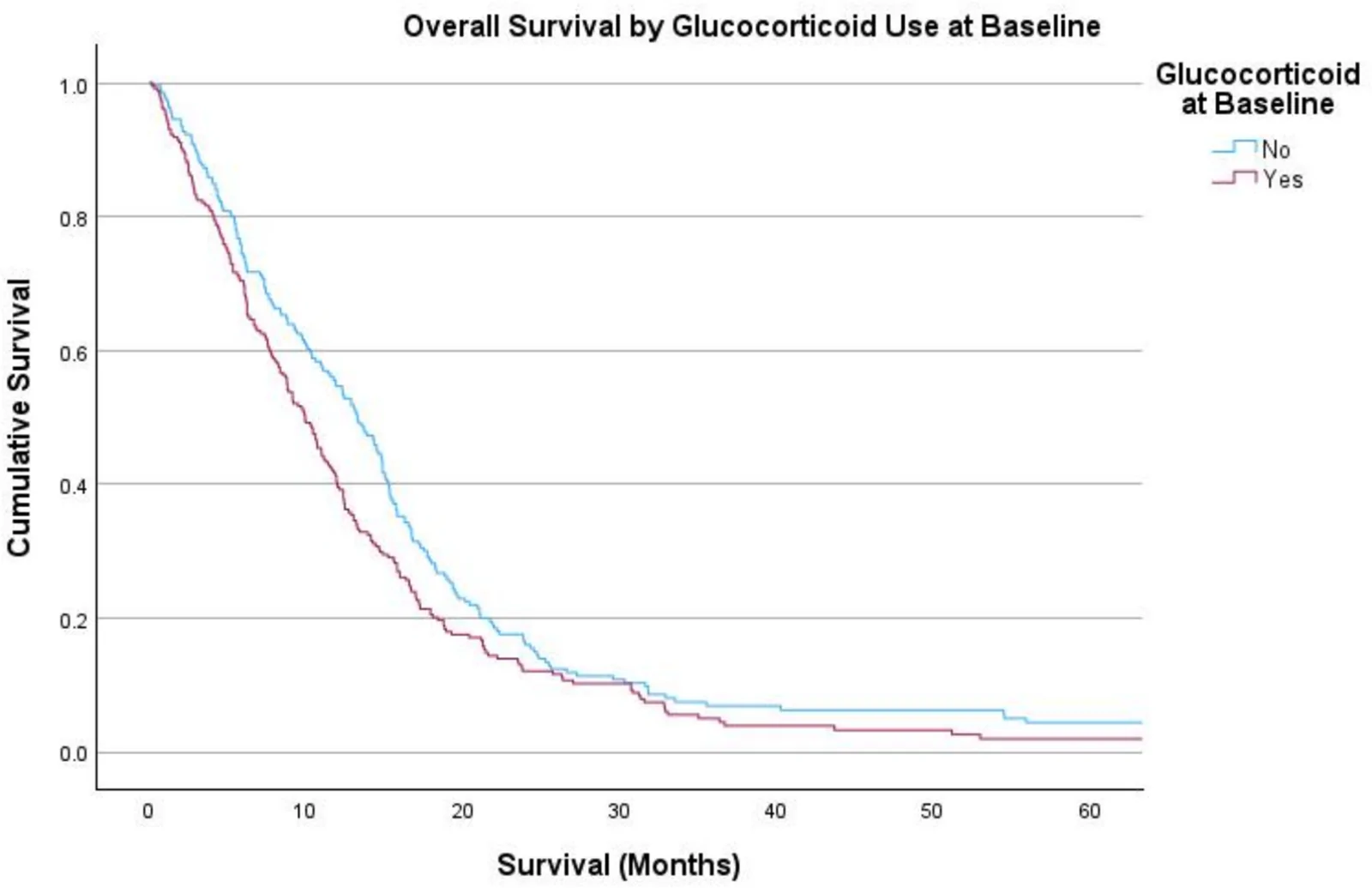
Kaplan–Meier Curve based on glucocorticoid use at baseline

**Fig. 7:**
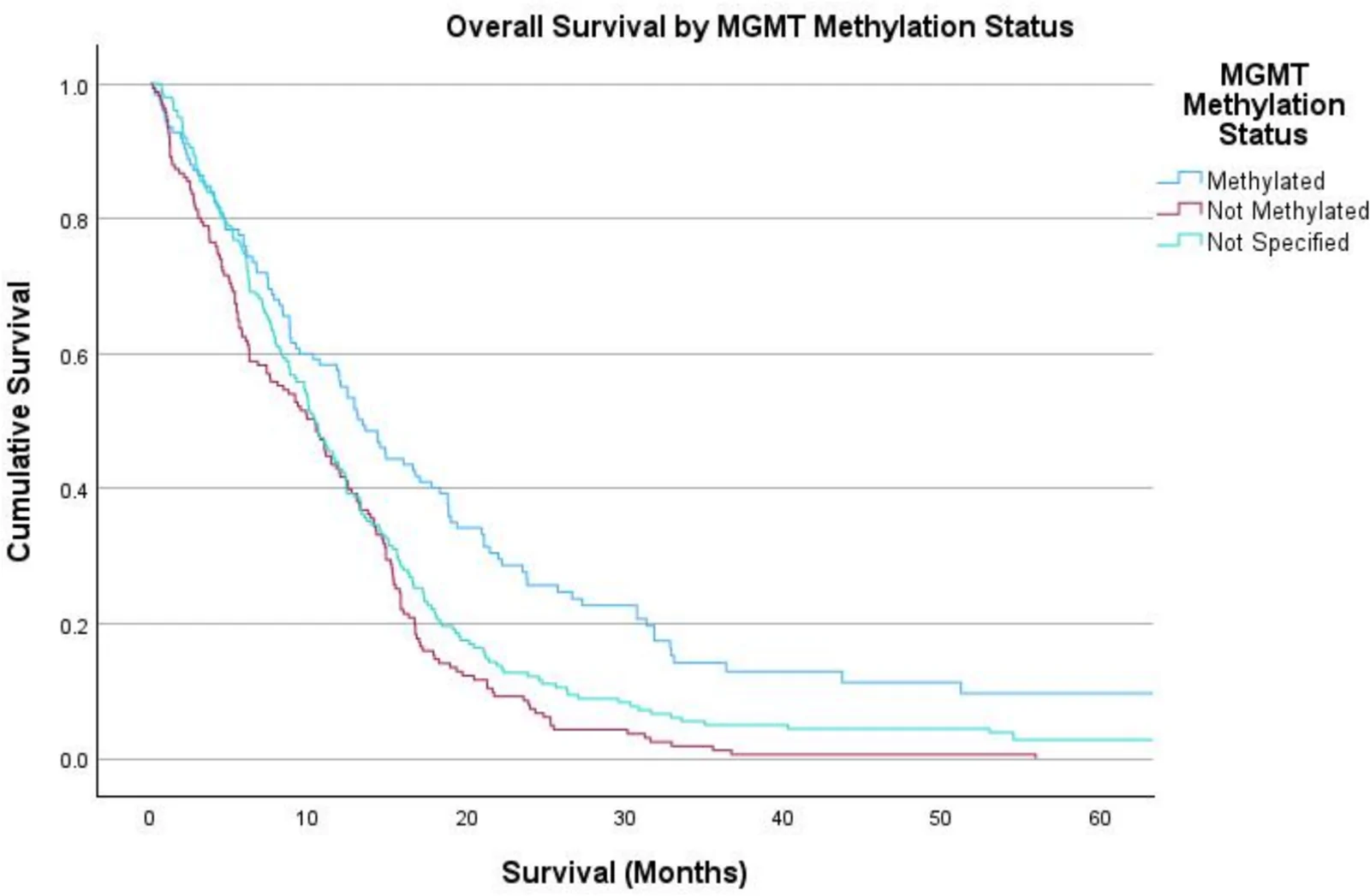
Kaplan–Meier Curve based on MGMT methylation status

**Fig. 8:**
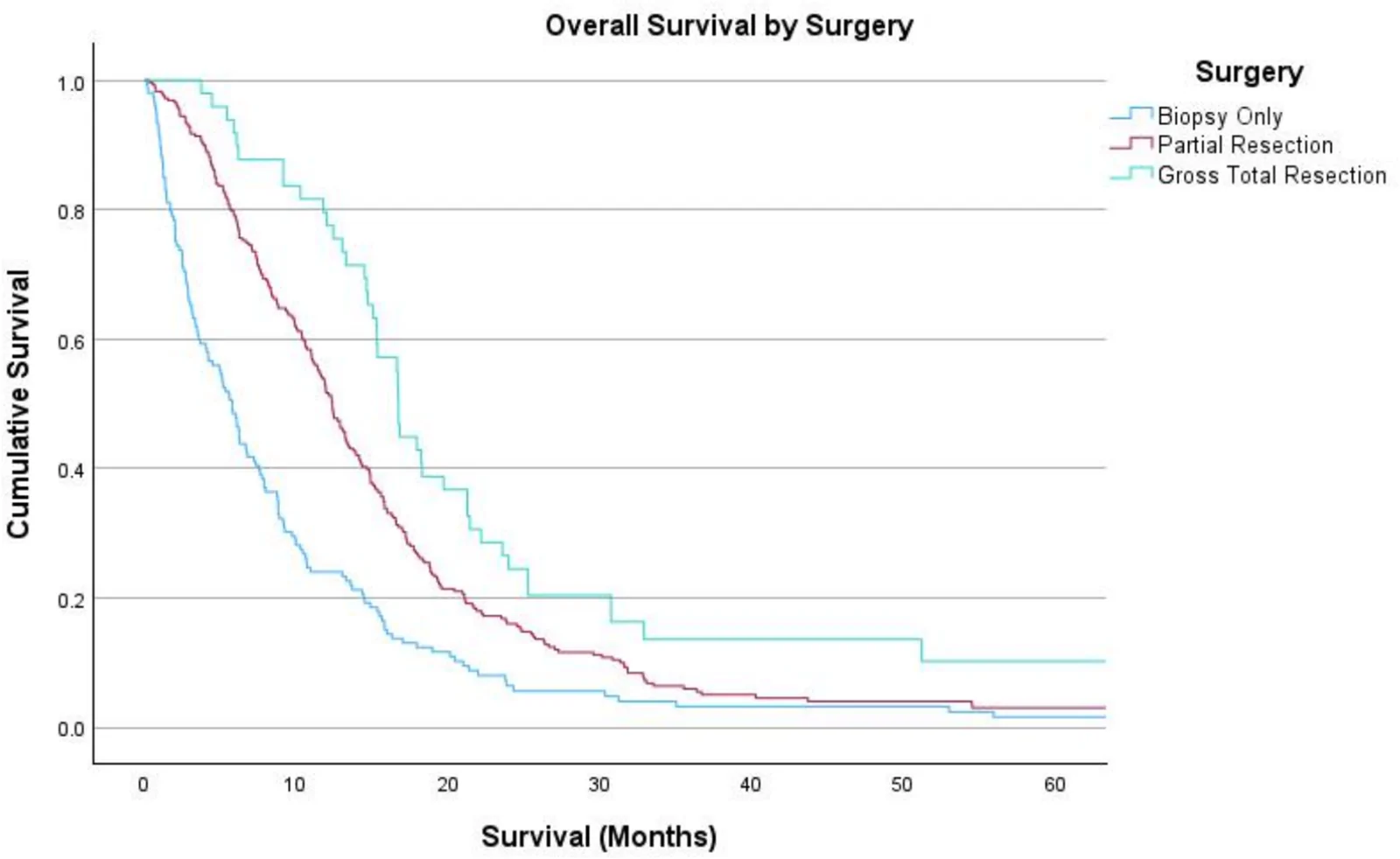
Kaplan–Meier Curve based on extent of surgical resection

**Fig. 9:**
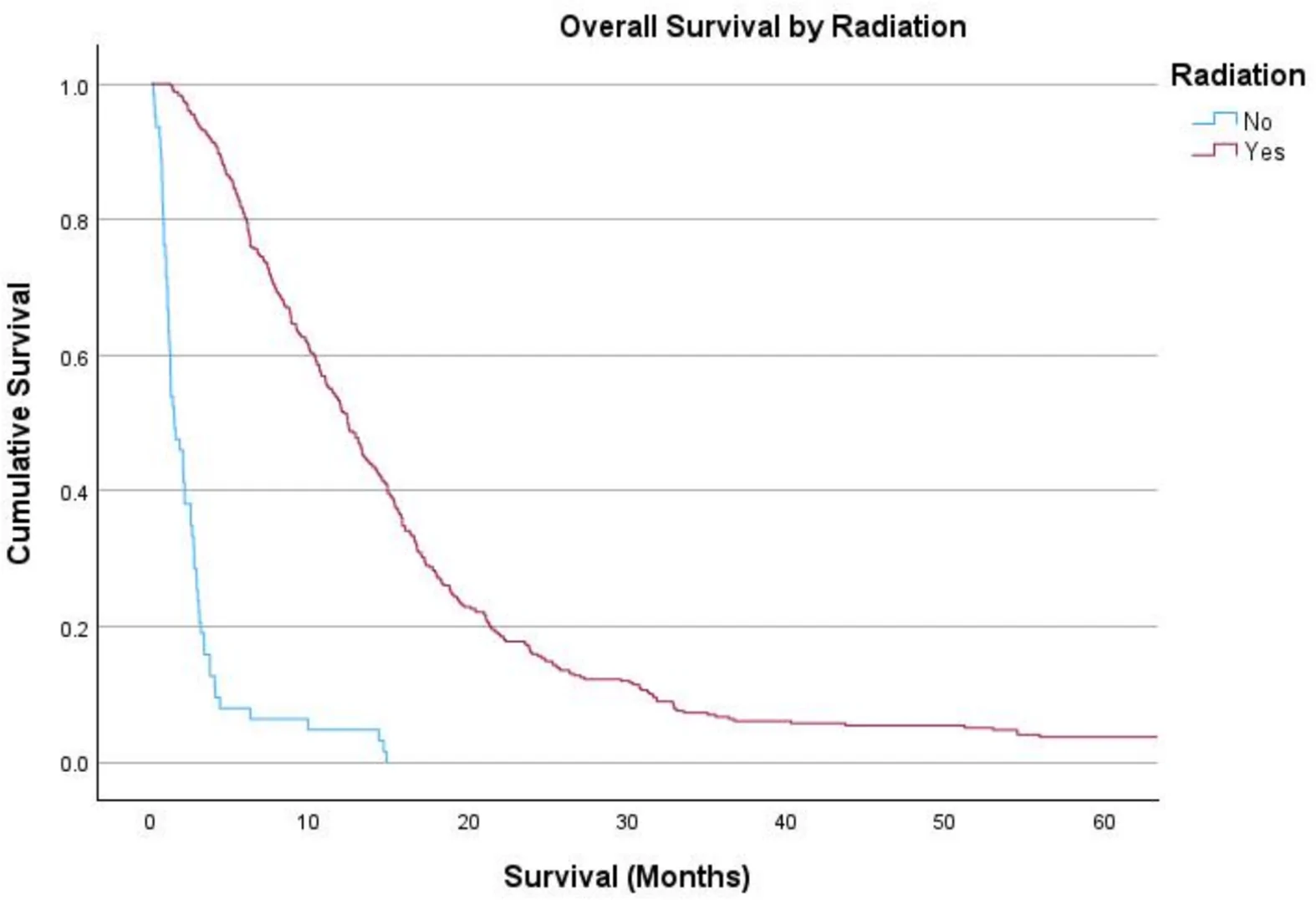
Kaplan–Meier Curve based on receipt of radiation

**Fig. 10:**
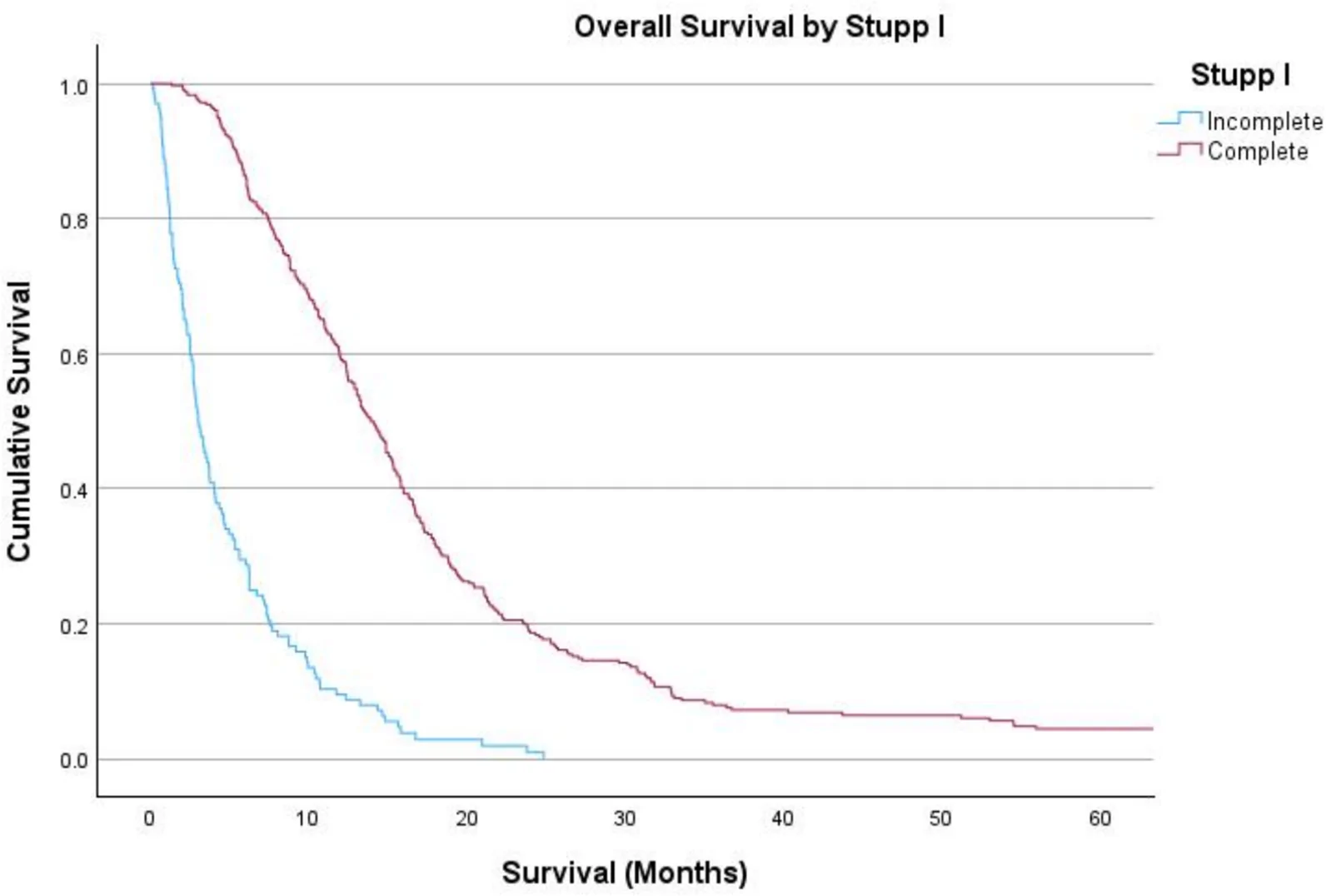
Kaplan–Meier Curve based on Stupp I protocol completion

**Fig. 11:**
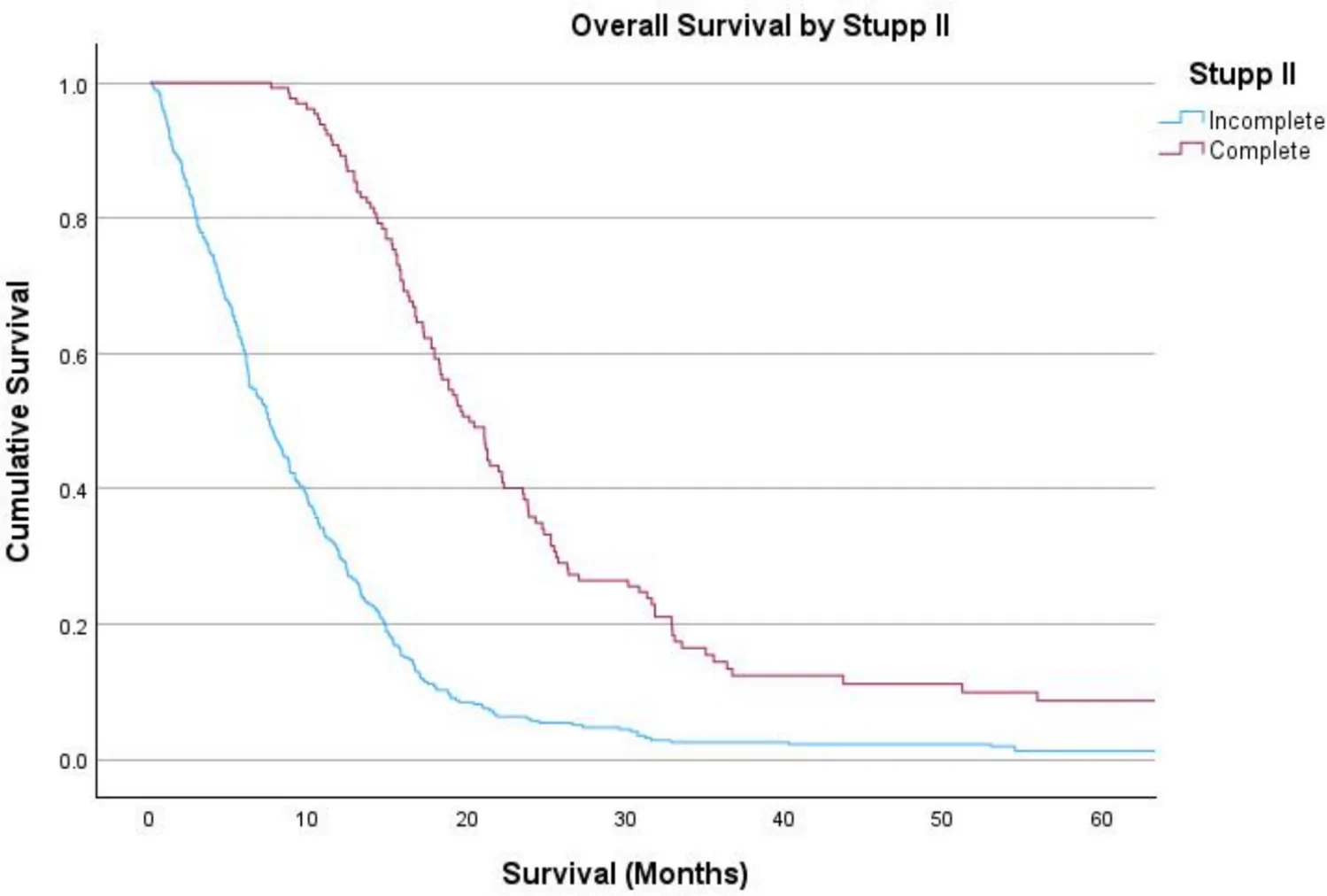
Kaplan–Meier Curve based on Stupp II protocol completion

**Fig. 12:**
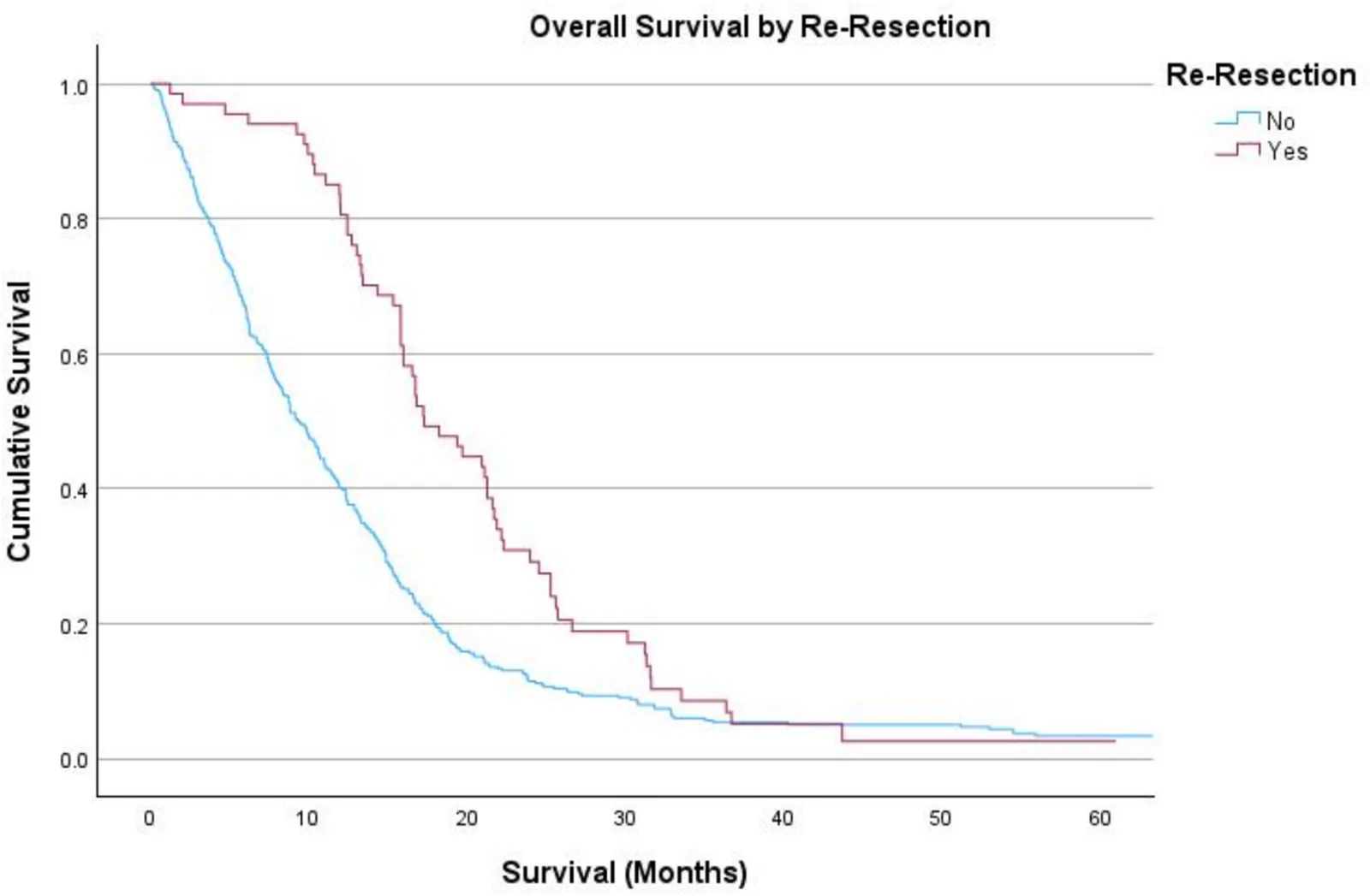
Kaplan–Meier Curve based on receipt of re-resection

**Fig. 13:**
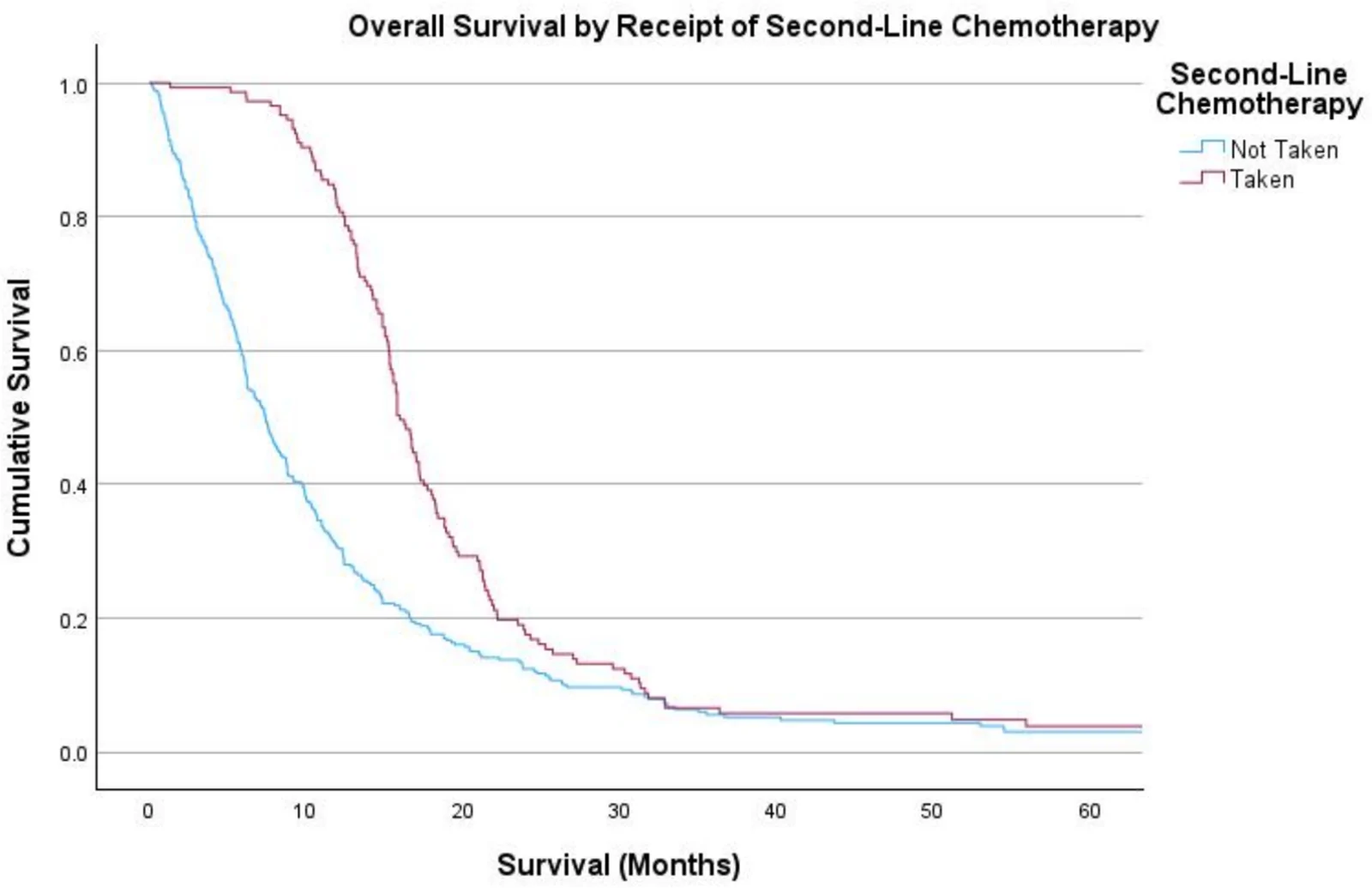
Kaplan–Meier Curve based on receipt of second line systemic therapy

Kaplan–Meier survival curves are shown in Figs. 1, 2, 3, 4, 5, 6, 7, 8, 9, 10, 11, 12 and 13, illustrating survival trends based on time period of diagnosis (Fig. 1), sex (Fig. 2), age at diagnosis (Fig. 3), ECOG performance status (Fig. 4), seizure history (Fig. 5), glucocorticoid use at baseline (Fig. 6), MGMT promoter methylation status (Fig. 7), extent of surgical resection (Fig. 8), receipt of radiation (Fig. 9), Stupp I protocol (Fig. 10), Stupp II protocol (Fig. 11), receipt of re-resection (Fig. 12) and receipt of second line chemotherapy (Fig. 13). Appendix A contains censored and uncensored curves for these figures.

The 5-year overall survival rate, calculated using the Kaplan–Meier method, was 2% for the overall cohort. Across the four diagnostic periods, the 5-year survival was 2.73% in 2006–2010, 1.09% in 2011–2014, 4.96% in 2015–2019, and 0% in 2020–2023. Among the MGMT promoter methylation subgroups, the 5-year survival rate was 4.8% for methylated patients and 0% for unmethylated patients. Patients who underwent gross total resection as the initial surgery had a 5-year survival rate of 6.1%.

Independent prognostic indicators for overall survival on multivariate analysis were age at diagnosis, ECOG performance status, glucocorticoid use at baseline, MGMT methylation status, receipt of radiation, and completion of the Stupp protocol (Table 2).

## Discussion

Despite significant contemporaneous advances in cancer outcomes for other cancer types [30], our study demonstrated no change in mOS in patients assessed at our cancer centre over a period of almost 2 decades. On multivariate analysis, key prognostic factors identified included age at diagnosis, ECOG performance status, glucocorticoid use at baseline, MGMT methylation status and completion of adjuvant chemoradiotherapy. However, in keeping with international real-world evidence, the mOS in our cohort has remained largely unchanged, highlighting the persistent international gap in progress between glioblastoma outcomes and those of other cancers [11, 31].

Our results mirror those of other studies as large-scale registry analyses have demonstrated the persistently poor survival associated with glioblastoma in real-world settings. In Swedish data from 1,458 adults with IDH-wildtype glioblastoma, the mOS was 345 days (~ 11.3 months) with a 2-year survival rate of 21.5% [32]. Similarly, Norwegian registry data from 1,158 adults reported an age-standardised mOS of 14.4 months for patients aged 18–70 years, with markedly lower survival in older patients [33]. Equally, despite including variable patient numbers ranging from 77 to 5392, several additional single institution or single country retrospective studies on patient outcomes have found similar real-world patient outcomes [7–12]. The mOS in our cohort (11.3 months) is comparable to the Swedish cohort and aligns with survival expectations for older or less fit patients in the Norwegian cohort. The mOS in our cohort of patients > 65 years of age (7.3 months) compared favourably with a Canadian BC Cancer Registry study of patients ≥ 60 years old (mOS 6.6 months) [34].

Regarding prognostic factors, age at diagnosis and ECOG performance status—strong predictors in both Swedish and Norwegian cohorts—were independently associated with survival in our study. In addition, we identified MGMT promoter methylation status, receipt of radiation, and completion of the Stupp protocol as significant independent predictors. These variables provide a more comprehensive survival model than previous registry analyses and emphasise actionable factors that may guide clinical management and treatment planning [35, 36].

Glucocorticoid exposure has also been identified as an adverse prognostic factor in glioblastoma. Prolonged or baseline steroid use has been associated with poorer outcomes, potentially due to immunosuppressive effects and reduced chemotherapeutic efficacy [17, 37, 38]. Our findings align with these reports, reinforcing the importance of minimising corticosteroid use where clinically feasible.

Consistent with these studies and others, our findings confirm that long-term survival remains extremely rare. The 5-year overall survival was only 2% across the cohort, with minimal differences observed across the four diagnostic periods (2.73% in 2006–2010, 1.09% in 2011–2014, 4.96% in 2015–2019, and 0% in 2020–2023). Subgroup analyses revealed slightly better outcomes among MGMT-methylated patients (4.8%) and those who underwent gross total resection (6.1%), highlighting small subsets of patients who may achieve longer-term survival.

Taken together, these comparisons underscore that despite evolving diagnostics, molecular characterisation, and therapeutic strategies, glioblastoma continues to carry a dismal prognosis in real-world populations. Our data complement international registry findings, highlighting the urgent need for improved therapies and interventions that can meaningfully impact long-term survival [29, 37, 38].

Evolution in our understanding of molecular alterations in high grade gliomas has led to more comprehensive molecular testing and altered diagnostic criteria [15]. As diagnostic standards transitioned from histopathological to integrated molecular definitions, the exclusion of IDH-mutant tumours from the glioblastoma category likely contributed to apparent survival shifts over time. Whether treatment options or data from trials based on previous histological diagnosis standards can be replicated in the current setting of molecularly diagnosed glioblastoma is an ongoing controversy in the field. Upcoming Data from the HISTO-MOL GBM collaborative group may help in this regard [39].

We observed a higher proportion of patients aged > 65 years and with ECOG performance status 2–4 referred to medical oncology in the later time periods, although these differences were not statistically significant, reflecting a shift in referral patterns over time. Additionally, a lower proportion of patients completed adjuvant Stupp phase I chemoradiotherapy in the later cohorts, declining from 80% in 2006–2010 and 79% in 2011–2014 to 67% in 2015–2019 and 68% in 2020–2023. We suspect these factors may have adversely impacted the overall mOS in this cohort, however multivariate analysis was not statistically significant either way. Similar demographic and performance-status differences have been observed in other registry studies, where older age and poor baseline function were dominant prognostic determinants [32, 33].

Unfortunately, many of the patients in the more recent cohort were diagnosed and managed during the COVID-19 pandemic, and shortly thereafter, a major cyberattack on the Irish healthcare system with all of the associated difficulties and delays in accessing treatment [40]. These systemic disruptions likely further contributed to treatment delays and suboptimal therapy delivery, paralleling international observations of pandemic-related care interruptions [41–45]. We suspect these led to additional unquantifiable impact on patient outcomes, however the numbers involved and nature of these factors meant we were unable to perform an adequately powered statistical analysis. A separate study evaluating the impact of both of these events on patient outcomes is planned.

Concordant with other studies [34, 46–49] we observed poorer outcomes in older patients. Management of this group is often heterogeneous due to the inability to receive aggressive resection, more prolonged radiotherapy courses, combined modality therapy due to impaired neurological and/or poor performance rates and the frequent presence of co-morbidities. More recently online tools have been developed including ones focusing on elderly patients to optimise decision making in the clinic and assist in prognostication and counselling [35].

TTF therapy has emerged as a recent adjunct to the standard Stupp protocol, providing incremental survival benefits in both trial and real-world settings. The EF-14 trial demonstrated a median overall survival improvement of approximately 5 months when TTFs were combined with Temozolomide [5]. Real-world analyses, such as those by Crompton et al., provides valuable insights into the real-world utilisation and efficacy of TTF for glioblastoma patients [26, 50]. Their multicentre analysis of 393 patients revealed that TTF, when added to standard external beam radiotherapy, was associated with improved two-year overall survival (58% vs. 41%, *p* < 0.006) and a trend towards decreased in-field failures. Despite these benefits, TTF adoption remained limited, with only 18.8% of patients receiving this treatment, highlighting barriers to widespread implementation [50]. Our study did not assess TTF utilisation, however TTF therapy was not reimbursed or widely available in Ireland during the time periods assessed.

In our cohort, the 5-year overall survival rate was 2%, with slightly better outcomes observed in MGMT-methylated patients (4.8%) and those undergoing gross total resection (6.1%). Integrating TTF into treatment regimens could potentially improve survival outcomes, particularly for patients with favourable prognostic factors. However, the low adoption rates observed by Crompton et al. underscore the need for strategies to overcome barriers to TTF implementation, such as cost, accessibility, and patient eligibility criteria [50]. Addressing the factors contributing to the limited adoption of TTF is crucial to ensure that its potential benefits are accessible to a broader patient population.

Ongoing improvements in the molecular characterisation of glioblastoma, and the development, approval, and timely use of novel therapies such as vaccine therapy, CAR-T cell therapy, small molecule immune-modulators, tyrosine kinase inhibitors, or other novel mechanisms for altering the tumor microenvironment and improving the efficacy of current therapies are needed to improve outcomes [31, 51–56]. Increased availability of high-throughput next generation sequencing (NGS) allows the identification of genetic alterations which have led to the incorporation of targeted therapies in the treatment of a broad array of malignancies [57]. However, the application of NGS testing in providing personalised cancer treatments for glioblastoma patients has proven challenging. At present, only the use of targeted therapy in BRAF p.V600E mutant recurrent CNS tumours is considered standard of care [58, 59]. Despite regular detection of a number of other molecular alterations in patients with glioblastoma the data to support targeted therapies in their presence is generally not robust [59]. Detection of high tumour mutational burden, microsatellite instability, PIK3CA mutations, FGFR mutations or EGFR mutations are well described and may provide therapeutic targets in the future [59, 60]. The increasing utilisation and evidence base regarding re-irradiation may improve outcomes for a small proportion of highly selected patients.

A key strength of this study lies in its comprehensive analysis over an extended period, which enabled the identification of evolving trends and shifts in glioblastoma management. None the less, the lack of improvement in outcomes despite improvements in diagnostics, multidisciplinary care, treatment resourcing and cancer outcomes in Ireland during this time period is disappointing [30, 61]. Our results contrast sharply with a similar analysis of contemporaneous outcomes for patients with non-small cell lung cancer at our centre where significant improved outcomes were observed [62]. At a clinical level our study highlights the need for integration of tools to guide decision making in elderly patients, early integration of palliative care supports for patients and their families, and prioritisation of clinical trial access for this tumour type [63].

The study has several limitations. First, given this is a single-centre retrospective study, it may be prone to selection bias. Since the study was conducted at a single institution and only amongst patients referred for a medical oncology assessment at our hospital, the patient population may not be representative of the general population, limiting the generalisability of the results to other settings or diverse patient groups. Secondly, the lack of IDH assessment in the earlier era limits some of the analysis in that cohort. A small number of patients in the earlier cohort (estimated to be 5–10%) were likely to have IDH mutations in their tumour which may have had a modest inflationary effect on the mOS data for this cohort [64].

## Conclusions

This study highlights the persistent challenges in improving real-world survival outcomes for patients with glioblastoma, despite major advances in molecular diagnostics and treatment protocols over the past two decades. Our findings reaffirm that age, performance status, MGMT methylation, and completion of adjuvant chemoradiotherapy remain key prognostic factors influencing survival.

Given the increasing proportion of older and frailer patients presenting with glioblastoma, future research should focus on strategies to improve treatment tolerance and optimise therapeutic outcomes in this group. These may include shorter-course radiotherapy regimens, dose-adjusted chemotherapy, supportive care interventions, and multidisciplinary approaches that integrate geriatric assessment into treatment planning.

The growing understanding of glioblastoma biology should also guide the transition towards precision medicine approaches. Broader integration of molecular profiling and next-generation sequencing could identify actionable alterations and support enrolment in biomarker-driven clinical trials. In particular, expanding access to emerging modalities—such as tumour-treating fields, vaccine therapy, CAR-T cell therapy, and targeted inhibitors—represents a critical step in enhancing survival and quality of life for selected patients.

By providing long-term, real-world data from a single tertiary cancer centre, our study contributes to the international evidence base demonstrating the gap between clinical trial efficacy and real-world outcomes. These findings may help inform the design of future clinical studies, with an emphasis on inclusive trial populations that better reflect the age, comorbidity burden, and treatment challenges seen in contemporary clinical practice.

## Data Availability

The datasets generated and/or analysed during the current study are not publicly available due to ethical restrictions protecting participant privacy but are available from the corresponding author on reasonable request.
